# Prediction of pre-eclampsia and its subtypes in high-risk cohort: hyperglycosylated human chorionic gonadotropin in multivariate models

**DOI:** 10.1186/s12884-018-1908-9

**Published:** 2018-07-03

**Authors:** Katja Murtoniemi, Pia M. Villa, Jaakko Matomäki, Elina Keikkala, Piia Vuorela, Esa Hämäläinen, Eero Kajantie, Anu-Katriina Pesonen, Katri Räikkönen, Pekka Taipale, Ulf-Håkan Stenman, Hannele Laivuori

**Affiliations:** 1 University of Helsinki and Turunmaa District Hospital, Gynaecological Outpatient Clinic, Hospital District of Southwest Finland, Kaskenkatu 13, 20700 Turku, Finland; 2 Obstetrics and Gynecology, University of Helsinki and Helsinki University Hospital, P.O. Box 140, FI-00029 Helsinki, Haartmaninkatu 2 Finland; 3 Department of Biostatistics, University of Turku, FI-20014 TURUN YLIOPISTO, Turku, Finland; 4 Department of Obstetrics and Gynaecology, Oulu University Hospital and University of Oulu, Kajaanintie 50, 90220 Oulu, Finland; 5 Finnish Medical Society Duodecim / Current Care, PL 713, Kalevankatu 11 A, 00101 HELSINKI, Finland; 6 HUSLAB and Department of Clinical Chemistry, University of Helsinki and Helsinki University Hospital, PO BOX 720, 00029 Helsinki, Finland; 7 Hospital for Children and Adolescents, University of Helsinki and Helsinki University Hospital, Stenbäckinkatu 11, P.O. Box 281, FI-00029 Helsinki, Finland; 8 Department of Psychology and Logopedics, University of Helsinki, Siltavuorenpenger 1-5, P.O. Box 9, FI-00014 Helsinki, Finland; 9 Suomen Terveystalo Oy, Asemakatu 22-24, 70100 Kuopio, Finland; 10 Institute for Molecular Medicine and Medical and Clinical Genetics, University of Helsinki, P.O. Box 63, FI-00014 Helsinki, Finland; 11 University of Helsinki and Helsinki University Hospital, P.O. Box 63, FI-00014 Helsinki, Finland

**Keywords:** Pre-eclampsia, Screening, Biomarkers, Early-onset pre-eclampsia, Late-onset pre-eclampsia, Severe pre-eclampsia, Placental growth factor, hCGβ, Hyperglycosylated human chorionic gonadotropin, Pregnancy associated plasma protein a

## Abstract

**Background:**

The proportion of hyperglycosylated human chorionic gonadotropin (hCG-h) to total human chorionic gonadotropin (%hCG-h) during the first trimester is a promising biomarker for prediction of early-onset pre-eclampsia. We wanted to evaluate the performance of clinical risk factors, mean arterial pressure (MAP), %hCG-h, hCGβ, pregnancy-associated plasma protein A (PAPP-A), placental growth factor (PlGF) and mean pulsatility index of the uterine artery (Uta-PI) in the first trimester in predicting pre-eclampsia (PE) and its subtypes early-onset, late-onset, severe and non-severe PE in a high-risk cohort.

**Methods:**

We studied a subcohort of 257 high-risk women in the prospectively collected Prediction and Prevention of Pre-eclampsia and Intrauterine Growth Restriction (PREDO) cohort. Multivariate logistic regression was used to construct the prediction models. The first model included background variables and MAP. Additionally, biomarkers were included in the second model and mean Uta-PI was included in the third model. All variables that improved the model fit were included at each step. The area under the curve (AUC) was determined for all models.

**Results:**

We found that lower levels of serum PlGF concentration were associated with early-onset PE, whereas lower %hCG-h was associated with the late-onset PE. Serum PlGF was lower and hCGβ higher in severe PE, while %hCG-h and serum PAPP-A were lower in non-severe PE. By using multivariate regression analyses the best prediction for all PE was achieved with the third model: AUC was 0.66, and sensitivity 36% at 90% specificity. Third model also gave the highest prediction accuracy for late-onset, severe and non-severe PE: AUC 0.66 with 32% sensitivity, AUC 0.65, 24% sensitivity and AUC 0.60, 22% sensitivity at 90% specificity, respectively. The best prediction for early-onset PE was achieved using the second model: AUC 0.68 and 20% sensitivity at 90% specificity.

**Conclusions:**

Although the multivariate models did not meet the requirements to be clinically useful screening tools, our results indicate that the biomarker profile in women with risk factors for PE is different according to the subtype of PE. The heterogeneous nature of PE results in difficulty to find new, clinically useful biomarkers for prediction of PE in early pregnancy in high-risk cohorts.

**Trial registration:**

International Standard Randomised Controlled Trial number ISRCTN14030412, Date of registration 6/09/2007, retrospectively registered.

**Electronic supplementary material:**

The online version of this article (10.1186/s12884-018-1908-9) contains supplementary material, which is available to authorized users.

## Background

Pre-eclampsia is a pregnancy-specific multisystem disorder, the pathogenesis of which is incompletely understood. It occurs in 2–8% of pregnancies and is one of the leading causes of maternal and fetal morbidity and mortality globally [1]. The early identification of women at high risk for pre-eclampsia would guide in planning of their follow-up during pregnancy and in the application of preventive measures. There is evidence that low-dose aspirin, started at 12–16 weeks of gestation, reduces the risk of pre-eclampsia [2, 3].

Clinical risk factors, e.g. antiphospholipid antibody syndrome, prior pre-eclampsia, chronic hypertension and pregestational diabetes have been used in early pregnancy in identifying those women at high risk of developing pre-eclampsia [4, 5]. A predictive test with high sensitivity and positive predictive value that incorporates maternal risk factors, biomarkers and biophysical measurements is needed to implement prophylaxis strategies [6]. Recently, we have shown, that serum hyperglycosylated human chorionic gonadotropin (hCG-h) is a promising marker of early-onset pre-eclampsia [7], but this was a case-control study. Thus, our aim in the present study was to test hCG-h or proportion of hCG-h to hCG (%hCG-h) in a cohort of high-risk women. We wanted to investigate if hCG-h could predict pre-eclampsia when combined with other biomarkers including serum free hCG beta (hCGβ), pregnancy-associated plasma protein-A (PAPP-A) and placental growth factor (PlGF), as well as biophysical measurements, mean arterial pressure (MAP) and Doppler ultrasound measurement of the mean pulsatility index of the uterine artery (Uta-PI). For this purpose, we constructed multivariate regression models and tested their predictive value to detect the different subtypes of pre-eclampsia.

## Methods

### Study cohort

The present study is a part of the multidisciplinary ‘Prediction and Prevention of Pre-eclampsia and Intrauterine Growth Restriction’ (PREDO) project [8]. A subcohort with known risk factor status for pre-eclampsia was prospectively collected between September 2005 and June 2009 in ten participating maternity clinics in Finland. The inclusion and exclusion criteria are described in Additional file 1: Table S1. Of the women with clinical risk factors for pre-eclampsia, 66 were excluded from the analyses because they took prophylactic aspirin during pregnancy as a part of a randomised trial [9]. Originally the subcohort of the present study comprised of 267 women. Ten women were not included to the analyses for various reasons: one woman had miscarriage at 19 weeks of gestation, one woman discontinued the study due to a non-medical reason and eight women were excluded due to missing data.

### Biophysical measurements

Gestational age was confirmed by first trimester ultrasound measurement of the fetal crown-rump length. Doppler ultrasound measurements were performed transvaginally at 11 0/7–13 6/7 weeks of gestation. The uterine artery flow was measured from the level of the inner os of the cervix, as it approaches the uterus laterally. The mean PI was calculated and then the multiple of the median (MoM) mean PI was calculated with the equation: Log_e_ mean Uta-PI = 1.39–0.012 × GA + GA^2^ × 0.0000198 MoM [10]*,* where GA = gestational age in days*.* The MAP was calculated from the first trimester visit blood pressure measurement with the equation: MAP = diastolic blood pressure + (systolic blood pressure – diastolic blood pressure)/3. The bilateral measurements of the Uta-PI were available for 83.7% (215/257) of high-risk women.

### Biomarkers

Fasting blood samples were drawn from antecubital veins at 11 0/7–13 6/7 (mean 13 0/7) weeks of gestation. Serum was separated within an hour by centrifugation and stored in − 80 °C until analysis. Serum hCG-h concentrations were determined using a time-resolved immunofluorometric assay. To eliminate the interaction between complement and B152 antibody in the hCG-h assay, serum samples were diluted 100-fold prior to analysis with EDTA-containing buffer (5 mmol/L) [11].

Serum hCG, hCGβ, PAPP-A and PlGF levels were measured using time-resolved immunofluorometric assay according to the manufacturer’s instructions (AutoDelfia, Perkin Elmer, Wallac, Turku, Finland). The intra- and inter-assay coefficients of variation were 4,9% (mean) and 6,7% (mean) for PlGF, < 2.3 and < 3.7% for PAPP-A, < 3.3 and < 6.2% for hCGβ, 1.8 and < 8.8% for hCG and 2.2 and < 10.5% for hCG-h, respectively.

### Outcome measures

Primary outcome was pre-eclampsia, defined as a systolic blood pressure ≥ 140 mmHg and/or a diastolic blood pressure ≥ 90 mmHg occurring after 20 weeks of gestation combined with a urinary 24-h protein excretion of ≥ 0.3 g or the dipstick equivalent in two consecutive measurements [12]. Secondary outcomes were: early-onset pre-eclampsia (diagnosed before 34 0/7 weeks of gestation), late-onset pre-eclampsia (diagnosed at or after 34 0/7 weeks of gestation), severe pre-eclampsia (systolic blood pressure ≥ 160 mmHg and/or diastolic blood pressure ≥ 110 mmHg and/or proteinuria ≥5 g/24 h), non-severe pre-eclampsia (pre-eclampsia not fulfilling the criteria of severe pre-eclampsia) and small for gestational age (SGA) (birthweight < − 2 standard deviations (SD) i.e. approximately < 2,5% percentile) [13]. All diagnoses were independently confirmed by a jury of two physicians and one research nurse, as described previously [9].

### Statistical analyses

Binary logistic regression was used to compare the characteristics of the study groups. The mean of continuous variables was calculated to compare the differences between the groups, if the variable was normally distributed. If the number of subjects in a subgroup was low or the continuous variable was not normally distributed, the median and interquartile range was reported.

The concentrations of hCG-h, hCG, hCGβ, PAPP-A, and PlGF, as well as %hCG-h were normally distributed after log-transformation and were adjusted for gestational age using linear regression analysis. The median regression equation was calculated from the respective concentrations measured from 107 women without clinical risk factors in the PREDO cohort and from a screening cohort from the Kuopio University Hospital [7]. Then, concentrations measured from women with clinical risk factors in the PREDO cohort were compared to the gestational age-adjusted median and expressed as MoM.

The binary logistic regression was used to evaluate univariate associations between measured variables and outcomes. The results were presented with odds ratios (OR). Since the number of investigated predictor variables was large compared to the number of women who developed pre-eclampsia, prediction models were built using regularised logistic regression with the L1/L2-norm using the R package glmnet [14]. Cross validation was used to select regularisation variables and separately to assess model fit. Three separate models were fitted for all outcomes on clinical bases. Maternal clinical background variables and MAP in the first trimester were included in model 1, since these variables are obtainable and economical. Biomarkers, which were the main interest of the study, were added in model 2 and MoM of the mean Uta-PI was added in model 3 to clarify if it still improves prediction rates. All variables that improved the model fit in the multivariate logistic regression analyses were included at each step, because we wanted to investigate the maximum prediction potential of each model. We used 10-time cross validation to compensate the lack of replication cohort.

The background variables used for model fitting were maternal age, primiparity, pre-pregnancy body mass index (BMI), infertility treatment before the present pregnancy, pre-eclampsia in a previous pregnancy, a SGA infant or gestational hypertension, type 1 diabetes mellitus and fetus mortus in a previous pregnancy. The area under the receiver operating characteristic (ROC) curve (AUC) value was determined for each model and models were compared using the R package pROC [15]. Results of multivariate logistic regression analyses are presented without confidence intervals, as confidence intervals obtained from regularised methods are problematic [16]. In addition to AUROC comparison, models were compared by calculating the *p*-values with DeLongs method. [17]. As our aim was to assess the screening performance of the models in clinical practice, the reference group was all women who did not develop the outcome of interest, as in the study by Kenny et al. [18]. For example, for severe pre-eclampsia the reference group consisted of women who did not develop severe pre-eclampsia: women without pre-eclampsia and women who developed non-severe pre-eclampsia.

Calculating study power is problematic regarding regularized logistic regression analyses used in present study, because power calculations are based on statistical significance and it is not possible to calculate significance with the statistical method used in this study. To give an indication of the power, power analysis was done for univariate logistic regression model. The power decreases when OR approaches 1, e.g. for pre-eclampsia (prevalence 13.2%, N 257) power is 96% with OR 0.5, 79% with OR 0.6, 49% with OR 0.7 and 22% with OR 0.8 [19].

## Results

### Baseline and pregnancy characteristics

The total cohort comprised of 257 women with risk factors for pre-eclampsia. Pre-eclampsia occurred in 34 (13.2%) of the women in the study cohort. Of those who developed pre-eclampsia, 9 (26.5%) had early-onset pre-eclampsia and 17 (50%) had a severe form of the disease. Clinical characteristics of the high-risk women who did or did not develop pre-eclampsia are presented in Table 1 and characteristics by pre-eclampsia subtype in Table 2.

**Table 1:** Characteristics of women with clinical risk factors for pre-eclampsia (PE), according to their PE status

	Women affected by PE (*N* = 34)	Women not affected by PE (*N* = 223)	p-value	OR^a^	95% CI Lower Upper	
Age, years (SD) ^b^	31.5 (5.8)	32.2 (5.7)	0.58	0.98	0.92	1.05
BMI, pre-pregnancy (kg/m^2^) (SD)^b^	29.7 (7.3)	28.1 (6.7)	0.19	1.04	0.98	1.09
Primiparous, n (%)	9 (26.5)	52 (23.2)	0.55	0.76	0.31	1.85
Infertility treatment, n (%)	4 (13.3)	24 (11.5)	0.88	0.91	0.28	2.99
Chronic disease, n (%)	18 (52.9)	93 (42.3)	0.67	0.84	0.38	1.88
Education, n (%)						
Elementary or less	3 (9.7)	4 (2.1)	0.04	4.98	1.06	23.44
High school or vocational school	8 (25.8)	57 (30.0)	0.64	0.81	0.34	1.92
Intermediate	14 (45.2)	81 (42.6)	0.79	1.12	0.52	2.38
University	6 (19.4)	48 (25.3)	0.49	0.71	0.28	1.83

^a^Binary logistic regression

^b^Mean

*OR* odds ratio, *BMI* body mass index, *CI* confidence interval, *SD* standard deviation,

**Table 2:** Characteristics of women with clinical risk factors for pre-eclampsia (PE) who developed PE, by subtype

Maternal Characteristics	Early-onset PE *n* = 9	Late-onset PE *n* = 25	*p*-value*	Severe PE *n* = 17	Non-severe PE n = 17	*p*-value*
Age, mean years (SD)	32.4 (4.9)	31.2 (6.1)	0.22	32.6 (5.5)	30.4 (6.0)	0.05
BMI, pre-pregnancy (kg/m^2^) (SD)	29.5 (8.1)	29.8 (7.1)	0.96	28.0 (7.2)	31.5 (7.1)	0.27
Primiparous, n (%)	2 (22.2)	2 (9.5)	1.00	7 (41.2)	2 (11.8)	0.02
Infertility treatment, n (%)	3 (33.3)	15 (60.0)	1.00	3 (17.6)	1 (7.7)	0.43
Chronic disease, n (%)	5 (56.6)	4 (16.0)	1.00	8 (47.1)	10 (58.8)	0.28

*Binary logistic regression

*BMI* body mass index, *SD* standard deviation

Early-onset PE = diagnosed before 34 0/7 weeks of gestation, late-onset PE = diagnosed at or after 34 0/7 weeks of gestation, severe PE = systolic blood pressure ≥ 160 mmHg and/or diastolic blood pressure ≥ 110 mmHg and/or proteinuria ≥5 g/24 h, Non-severe PE = PE not fulfilling the criteria of severe pre-eclampsia

Twelve (4.7%) women gave birth to a SGA newborn. Of these women, eight (67%) had developed pre-eclampsia. The prevalence of each inclusion criterion in women who developed pre-eclampsia and in women who did not develop pre-eclampsia is presented in Additional file 2: Table S2. There was one stillbirth at the 27th week of gestation. Pregnancy characteristics are presented in Table 3 and the median or mean values of measured variables in Additional file 3: Table S3.

**Table 3:** Pregnancy characteristics

Pregnancy characteristics of high-risk women	Women not affected by PE (*N = 223*)	PE (*N = 34*)	Late-onset PE (*n= 25*)	Early-onset PE (*n=9*)	Non-Severe PE (*n=17*)	Severe PE (*n=17*)
Antihypertensive medication n (%)	17 (7.6)	22 (64.7)	16 (64.0)	6 (66.7)	8 (47.1)	14 (82.4)
Before 20 weeks of gestation	5 (2.2)	2 (5.9)	2 (8.0)	0	1 (5.9)	1 (5.9)
After 20 weeks of gestation	12 (5.5)	20 (58.8)	14 (56.0)	6 (66.7)	7 (41.2)	13 (76.5)
Weight gain during pregnancy, kg	12.8 (6.7)	12.4 (6.6)	11.0 (4.0)	11.1 (10.0)	12.8 (6.6)	12.0 (8.6)
Gestational diabetes, n (%)	56 (25.2)	9 (26.5)	7 (28.0)	2 (22.2)	4 (23.5)	5 (29.4)
Oral glucose tolerance test not performed	17 (7.6)	2 (5.9)	1 (4.0)	1 (11.1)	1 (5.9)	1 (5.9)
Highest systolic blood pressure, mmHg	136 (26)	168 (28)	165 (24)	183 (27)	161 (20)	182 (27)
Highest diastolic blood pressure, mmHg	90 (17)	110 (8)	110 (15)	105 (14)	10 (13)	111 (10)
Highest proteinuria, g/day	0.2 (0.0)	1.2 (1.8)	0.8 (1.3)	2.4 (5.2)	0.7 (0.9)	1.9 (3.1)
Gestational weeks at birth	39.9 (6.8)	38.4 (6.6)	38.7 (1.6)	31.9 (6.2)	38.6 (2.0)	36.6 (7.2)
Mode of delivery, n (%)						
Vaginal	151 (68.6)	18 (52.9)	16 (64.0)	2 (22.2)	11 (64.7)	8 (41.2)
Vacuum extraction	20 (9.1)	1 (2.9)	1 (4.0)	0	0	0
Elective caesarean section	17 (7.7)	1 (2.9)	1 (4.0)	0	1 (8.3)	0
Caesarean section during labour	31 (14.2)	14 (41.2)	7 (28.0)	7 (77.8)	5 (29.4)	9 (52.9)
Apgar score at 5 min	9 (0)	9 (2)	9 (1)	8 (1)	9 (1)	8.5 (2)
Umbilical artery pH	7.26 (0.10)	7.25 (0.10)	7.25 (0.14)	7.27 (0.20)	7.24 (0.13)	7.25 (0.16)
Newborn birthweight, g	3615 (685)	3109 (1260)	3370 (663)	1350 (1240)	3570 (629)	2340 (1890)
Placental weight, g	610 (180)	545 (185)	580 (335)	350 (40)	570 (105)	440 (215)

With continuous variables median and interquartile range in parenthesis is presented

n = number of cases, PE = pre-eclampsia

Early-onset PE = pre-eclampsia diagnosis < 34 weeks of gestation; Late-onset PE = pre-eclampsia diagnosis ≥ 34 weeks of gestation; Severe pre-eclampsia = systolic blood pressure ≥ 160 mmHg and/or diastolic blood pressure ≥ 110 mmHg and/or proteinuria ≥5 g/24 h, Non-severe pre-eclampsia = PE not fulfilling the criteria of severe pre-eclampsia

### Univariate analyses

All results of univariate analyses are summarised in Table 4. None of the biomarkers were different between pre-eclamptic and non-pre-eclamptic women. The median Uta-PI and MAP were higher in women who developed pre-eclampsia than in women who did not develop pre-eclampsia. Women who developed early-onset pre-eclampsia had lower median PlGF and higher MAP compared to women who did not develop early-onset pre-eclampsia. Women who developed late-onset pre-eclampsia had lower %hCG-h and higher MAPs than other women. Median hCGβ, Uta-PI and MAP were higher, and median PlGF lower, in women with severe pre-eclampsia compared to other women. In women with non-severe pre-eclampsia, both the median PAPP-A and %hCG-h levels were lower than in other women.

**Table 4:** Results of the univariate analyses

Variable	Participants		Median (IQR) / Mean (SD)		OR (95% CI)	*p*-value
	PE, n	No PE, n	PE	No PE		
hCG-h MoM	34	223	0.27 (0.11–0.61)	0.30 (0.15–055)	0.98 (0.49–1.97)	0.958
%hCG-h MoM	34	223	0.77 (0.64–1.24)	0.99 (0.71–1.45)	0.55 (0.26–1.14)	0.107
hCGβ MoM	34	223	0.60 (0.35–0.89)	0.56 (0.35–0.91)	1.21 (0.70–2.10)	0.503
PAPP-A MoM	34	222	0.69 (0.45–0.92)	0.84 (0.52–1.26)	0.50 (0.24–1.05)	0.069
PlGF MoM	34	222	0.82 (0.65–1.06)	0.99 (0.81–1.22)	0.32 (0.10–1.00)	0.051
Uta-PI MoM	29	186	1.15 (0.83–1.25)	0.92 (0.71–1.12)	3.81 (1.12–12.95)	0.032
MAP^a^	34	211	102.1 (10.9)	94.7 (14.4)	1.05 (1.02–1.09)	0.001
Variable	EOPE, n	No EOPE, n	EOPE	No EOPE	OR (95% CI)	p-value
hCG-h MoM	9	248	0.61 (0.23–0.75)	0.29 (0.14–0.53)	0.65 (1.61–3.96)	0.304
%hCG-h MoM	9	248	1.32 (1.15–1.50)	0.92 (0.68–1.39)	1.58 (0.57–4.39)	0.381
hCGβ MoM	9	248	0.84 (0.61–1.04)	0.56 (0.34–0.91)	0.61 (1.41–3.26)	0.417
PAPP-A MoM	9	247	0.56 (0.50–0.85)	0.83 (0.51–1.21)	0.11 (0.46–2.01)	0.305
PlGF MoM	9	247	0.63 (0.47–0.73)	0.98 (0.80–1.22)	< 0.001 (< 0.001–0.04)	< 0.001
Uta-PI MoM	6	209	1.17 (0.97–1.25)	0.93 (0.73–1.14)	0.59 (5.56–52.2)	0.134
MAP^a^	9	236	104.3 (15.0)	95.4 (11.5)	1.06 (1.01–1.12)	0.028
Variable	LOPE, n	No LOPE, n	LOPE	No LOPE	OR (95% CI)	p-value
hCG-h MoM	25	232	0.20 (0.10–0.33)	0.30 (0.15–0.57)	0.26 (0.70–1.87)	0.478
%hCG-h MoM	25	232	0.76 (0.64–0.89)	1.02 (0.71–1.46)	0.30 (0.11–0.81)	0.018
hCGβ MoM	25	232	0.54 (0.31–0.79)	0.57 (0.35–0.91)	0.56 (1.09–2.13)	0.796
PAPP-A MoM	25	231	0.72 (0.43–0.95)	0.84 (0.51–1.22)	0.23 (0.54–1.24)	0.144
PlGF MoM	25	231	0.86 (0.80–1.18)	0.98 (0.80–1.21)	0.91 (0.34–2.41)	0.846
Uta-PI MoM	23	192	1.06 (0.78–1.33)	0.94 (0.72–1.13)	2.96 (0.78–11.23)	0.110
MAP^a^	25	220	101.3 (14.5)	95.0 (11.2)	1.04 (1.01–1.08)	0.013
Variable	Severe PE, n	No Severe PE, n	Severe PE	No Severe PE	OR (95% CI)	p-value
hCG-h MoM	17	240	0.47 (0.12–0.75)	0.30 (0.15–0.53)	1.63 (0.82–3.27)	0.165
%hCG-h MoM	17	240	0.90 (0.64–1.50)	0.95 (0.69–1.39)	0.94 (0.39–2.25)	0.885
hCGβ MoM	17	240	0.85 (0.35–1.13)	0.56 (0.35–0.90)	1.78 (1.01–3.16)	0.048
PAPP-A MoM	17	239	0.77 (0.45–1.08)	0.83 (0.51–1.21)	0.81 (0.37–1.80)	0.612
PlGF MoM	17	239	0.80 (0.61–0.83)	0.98 (0.80–1.22)	0.11 (0.02–0.69)	0.018
Uta-PI MoM	17	239	1.21 (0.96–1.43)	0.93 (0.73–1.13)	6.97 (1.4–34.2)	0.017
MAP^a^	17	228	105.8 (16.0)	94.9 (11.0)	1.08 (1.03–1.12)	0.001
Variable	Non-severe PE, n	No Non-severe PE, n	Non-severe PE	No Non-severe PE	OR (95% CI)	p-value
hCG-h MoM	17	240	0.15 (0.30–0.57)	0.22 (0.10–0.33)	0.22 (0.03–1.55)	0.127
%hCG-h MoM	17	240	0.78 (0.68–0.89)	0.98 (0.69–1.46)	0.28 (0.09–0.95)	0.041
hCGβ MoM	17	240	0.56 (0.37–0.75)	0.57 (0.35–0.92)	0.41 (0.10–1.63)	0.207
PAPP-A MoM	17	239	0.55 (0.49–0.90)	0.84 (0.51–1.26)	0.25 (0.07–0.94)	0.041
PlGF MoM	17	239	0.86 (0.73–1.08)	0.98 (0.80–1.21)	0.76 (0.22–2.65)	0.663
Uta-PI MoM	15	200	1.02 (0.82–1.21)	0.94 (0.73–1.14)	1.57 (0.30–8.31)	0.596
MAP^a^	17	228	98.3 (12.0)	95.5 (11.7)	1.02 (0.98–1.06)	0.336

^a^mean and SD

Binary logistic regression was used to evaluate the association of measured variables to pre-eclampsia and its subtypes

*IQR* interquartile range; *SD* standard deviation; *CI* confidence interval; *PE* pre-eclampsia; *EOPE* early-onset pre-eclampsia (diagnosed before 34 0/7 weeks of gestation); *LOPE* late-onset pre-eclampsia (diagnosed at or after 34 0/7 weeks of gestation); *BMI* body mass index; *SGA* small for gestational age; *DM* diabetes mellitus; *MAP* mean arterial pressure; *Uta-PI* pulsatility index of the uterine artery; *hCG* human chorionic gonadotropin; *hCG-h* hyperglycosylated hCG; *%hCG-h* the ratio of hCG-h to hCG; *PAPP-A* pregnancy-associated plasma protein a; *PlGF* placental growth factor; *MoM* multiple of the median

Early-onset PE = pre-eclampsia diagnosis < 34 weeks of gestation; Late-onset PE = pre-eclampsia diagnosis ≥ 34 weeks of gestation; Severe pre-eclampsia = systolic blood pressure ≥ 160 mmHg and/or diastolic blood pressure ≥ 110 mmHg and/or proteinuria ≥5 g/24 h, Non-severe pre-eclampsia = PE not fulfilling the criteria of severe pre-eclampsia

### Multivariate logistic regression models

The predictive models were constructed for pre-eclampsia and its subtypes as outcomes. All variables that improved the model fit in the multivariate logistic regression analyses were included, therefore each regression model is individual: for example model 1 for early-onset pre-eclampsia consists of different variables than model 1 for late-onset pre-eclampsia. The multivariate models, AUC values and diagnostic characteristics of pre-eclampsia and its subtypes are presented in Table 5.

**Table 5:** Multivariate regression models and screening test characteristics for pre-eclampsia and its subtypes

Outcome and model	Variables in model	Prevalence, %	AUC	Valid.AUC	At 90% Specificity for Validated AUC				At 95% Specificity for Validated AUC			
PE		13.2			Sensitivity, %	PPV, %	NPV, %	PLR	Sensitivity, %	PPV, %	NPV, %	PLR
Model 1	Age, prior PE, prior SGA, DM type-I, MAP		0.70	0.55	23	27	88	2.4	13	29	88	2.6
Model 2	Model 1 variables + hCG MoM, %hCG-h MoM, free beta hCG MoM, PAPP-A MoM, PlGF MoM		0.79	0.60	20	24	88	2.0	17	33	88	3.2
Model 3	Model 1 variables + CH, hCG MoM, %hCG-h MoM, free beta hCG MoM, PlGF MoM, Uta-PI MoM		0.85	0.66	36	36	90	3.7	16	33	88	3.3
EOPE		3.5										
Model 1	Primiparity, CH, prior SGA, DM type-I, MAP		0.84	0.52	7	10	86	0.7	6.7	17	87	1.3
Model 2	Primiparity, CH, DM type-I, MAP, hCG MoM, %hCG-h MoM, free, PlGF MoM		0.96	0.68	20	24	88	2.0	20	38	88	3.9
Model 3	Primiparity, prior PE, prior SGA, CH, MAP, hCG MoM, PlGF MoM		0.95	0.62	11	14	87	1.1	11	25	88	2.2
LOPE		9.7										
Model 1	Age, prior PE, prior SGA, CH, DM type-I, MAP		0.75	0.54	10	14	87	1.0	6.7	17	87	1.3
Model 2	Age, prior PE, prior SGA, CH, MAP, %hCG-h MoM, free beta hCG MoM		0.84	0.62	27	30	89	2.7	6.7	17	87	1.3
Model 3	Model 1 variables, prior FM, hCG MoM, %hCG-h MoM, free beta hCG MoM, PlGF MoM, Uta-PI MoM		0.89	0.66	32	33	90	3.3	16	33	88	3.3
Severe PE		6.6										
Model 1	MAP		0.68	0.58	20	24	88	2.0	17	33	88	3.2
Model 2	MAP, hCG MoM, free beta hCG MoM, PlGF MoM		0.78	0.62	23	27	88	2.4	23	41	89	4.5
Model 3	MAP, prior FM, hCG MoM, %hCG-h MoM, PlGF MoM, Uta-PI MoM		0.86	0.65	24	27	89	2.5	20	38	89	4.1
Non-Severe PE		6.6										
Model 1	Prior PE		0.71	0.54	3.3	5.6	86	0.4	3.3	7.1	86	0.5
Model 2	Age, BMI, prior PE, prior SGA, CH, %hCG-h MoM, PAPP-A MoM		0.86	0.58	17	21	88	1.7	10	23	87	1.9
Model 3	Age, BMI, prior PE, prior SGA, CH, DM type-I, prior FM, %hCG-h MoM, free beta hCG MoM, PlGF MoM, Uta-PI MoM		0.89	0.60	22	25	88	2.2	15	31	88	2.9

*Valid*., validated*; PPV*, positive predictive value*; NPV*, negative predictive value; *PLR*, positive likelihood ratio; *PE*, pre-eclampsia*; EOPE*, early-onset PE; *LOPE*, late-onset PE;

*BMI*, body mass index; *SGA*, small for gestational age; *FM*, fetus mortus; *DM*, diabetes mellitus; *CH,* chronic hypertension; *MAP*, mean arterial pressure; Uta-PI, pulsatility index of the uterine artery; *hCG*, human chorionic gonadotropin; *hCG-h*, hyperglycosylated hCG; %hCG-h, the ratio of hCG-h to hCG; *PAPP-A*, pregnancy-associated plasma protein A;

*PlGF*, placental growth factor; MoM, multiple of the median.

Early-onset PE = pre-eclampsia diagnosis < 34 weeks of gestation; Late-onset PE = pre-eclampsia diagnosis ≥ 34 weeks of gestation; Severe pre-eclampsia = systolic blood pressure ≥ 160 mmHg and/or diastolic blood pressure ≥ 110 mmHg and/or proteinuria ≥5 g/24 h, Non-severe pre-eclampsia = PE not fulfilling the criteria of severe pre-eclampsia.

Prediction models were built using regularised logistic regression. Cross validation was used to select regularisation variables and separately to assess model fit. The AUC values are expressed before and after 10-fold cross validation. Three separate models were fitted for all outcomes. First, background variables and MAP in the first trimester were included in the model (model 1). Next, biomarkers were added (model 2). Finally, MoM of the mean Uta-PI was added (model 3). All variables that improved the model fit in the multivariate logistic regression analyses were included.

The effect of each risk factor on the risk of developing pre-eclampsia was estimated from the model 1. The most significant factor that increased the risk of developing all pre-eclampsia (OR 1.69) and the late-onset (OR 2.40) or the non-severe (OR 2.32) subtypes was a history of pre-eclampsia. Primiparity (OR 3.34) was the most significant factor for the early-onset subtype.

For all pre-eclampsia, the best validated AUC value of 0.66 at sensitivities of 36 and 16% were achieved with 90 and 95% specificity, respectively, when combining maternal characteristics, MAP, biomarkers and Uta-PI MoM. With this model, positive predictive value (PPV) was 33% and negative predictive value was 88%.

The best multivariate model for prediction of early-onset pre-eclampsia was achieved by combining maternal characteristics, MAP and biomarkers. The validated AUC value was 0.68 with 20% sensitivity at both 90 and 95% specificity. The PPV for early-onset pre-eclampsia was 25% and NPV was 88%.

For prediction of late-onset pre-eclampsia, model 3 gave the highest prediction rates with an AUC value of 0.66, with 32 and 16% sensitivity at 90 and 95% specificity, respectively. For prediction of severe pre-eclampsia, the best validated AUC value was 0.65 with 24% sensitivity at 90% specificity. It was achieved by combining MAP, the a priori risk factor of having a previous fetus mortus, biomarkers and Uta-PI MoM (model 3). A sensitivity of 23% at 95% sensitivity was achieved by combining MAP and biomarkers (model 2). The best multivariate model for predicting non-severe pre-eclampsia, with a validated AUC value of 0.60, 22 and 15% sensitivity at 90 and 95% specificity, respectively, was attained with model 3.

## Discussion

To our knowledge, this is a first study investigating first trimester hCG-h as a potential pre-eclampsia predictor in a prospectively recruited high-risk cohort. In univariate analyses, %hCG-h levels were lower in women with late-onset and non-severe pre-eclampsia. Lower levels of serum PlGF were associated with early-onset and severe pre-eclampsia, and lower serum PAPP-A levels with non-severe pre-eclampsia. Higher serum hCGβ levels were associated with severe pre-eclampsia. The first trimester MAP was higher in pre-eclampsia and its subtypes, except in the non-severe subtype, when compared to all other participants. Uta-PI MoM was higher in women who developed pre-eclampsia compared to women who did not, and in women who developed severe pre-eclampsia compared to all other participants. Despite the abovementioned differences in serum %hCG-h, PlGF, PAPP-A, hCGβ, MAP and Uta-PI between the groups, multivariate models gave only a modest prediction of the disease and did not meet the requirement of a clinically useful screening test.

Pre-eclampsia in a previous pregnancy was the most important risk factor associated with pre-eclampsia in a subsequent pregnancy. This is in line with a recent meta-analysis, wherein women with prior pre-eclampsia had the greatest pooled relative risk for developing pre-eclampsia [5]. Interestingly, in our study, prior pre-eclampsia had the strongest association with late-onset and non-severe pre-eclampsia, whereas primiparity had the highest OR for early-onset pre-eclampsia in multivariate analyses. There are a few studies that distinguish between pre-eclampsia subtypes [20, 21]. In contrast to our study, other studies have been conducted in unselected populations. In accordance with our results, in the study of Odegård et al., prior pre-eclampsia and primiparity had the highest ORs for predicting pre-eclampsia, but primiparity did not appear to be specifically associated with either of the clinical subtypes [21].

The impaired invasion of cytotrophoblasts into the spiral arteries is thought to be one pathophysiological mechanism of pre-eclampsia [22]. The exact role of hCG-h in the pathophysiology of pre-eclampsia is not known. However, there is strong evidence that extravillous cytotrophoblasts initiate the production of hCG-h during their differentiation from proliferative cytotrophoblasts to invasive cytotrophoblasts in normal pregnancies and that hCG-h circulating in maternal serum reflects the invasion process of trophoblasts during the first trimester [23]. Thus, hCG-h or its ratio to total hCG might represent a biomarker of early placentation [24]. Lower %hCG-h in late-onset and non-severe forms of pre-eclampsia may indicate a mild developmental insufficiency of the placenta [25]. The results of the present study stand in contrast to a previous report, in which %hCG-h was lower in early-onset pre-eclampsia [7]. One possible explanation is that the median gestational age at the time of sampling was higher in this study (13.0 vs. 10.3 gestational weeks). In very early pregnancy (at 4 to 5 weeks of pregnancy) virtually all (90–100%) of the hCG in serum consists of hCG-h. The concentration decreases to 5–10% at 10 weeks and to 3% after 20 weeks [11, 24]. In the present study, the regression line reflecting median %hCG-h against gestational weeks in the scatterplot showed lower MoM values in early-onset pre-eclampsia before 12 weeks gestation than in the other groups. This is in agreement with previous publications [7, 26]. However, only nine women had early-onset pre-eclampsia. Thus, it is not possible to draw any definitive conclusions.

Adding the Doppler measurement of Uta-PI to the multivariate regression models increased the AUCs of the models (from model 2 to model 3) for all pre-eclampsia and its subtypes except for early-onset pre-eclampsia. This stands in contrast to earlier studies conducted in high-risk cohorts, where Uta-PI predicted early-onset pre-eclampsia and severe pre-eclampsia better than all pre-eclampsia or late-onset pre-eclampsia. However, our results are in accordance to the results from the same studies showing that mean Uta-PI is much less useful for prediction of pre-eclampsia and its subtypes in the first trimester in a high-risk population than in a low-risk or screening population [27–29].

### Strength and limitations

It should be noted that the definition of early-onset pre-eclampsia was different in the present study than in most studies. We defined early-onset pre-eclampsia as cases diagnosed before 34 weeks of gestation, whereas the definition in most of other studies is pre-eclampsia requiring delivery before 34 weeks of gestation.

Strength of our study is a carefully characterised, prospectively collected cohort of women with clinical risk factors for pre-eclampsia. A jury of two physicians and one research nurse independently confirmed all pre-eclampsia diagnoses. Furthermore, our prospective study reflects the true incidence of early-onset and late-onset pre-eclampsia in high-risk women.

A limitation of our study is the relatively small sample size, but the high incidence of PE cases (13.2%) allowed us to obtain some interesting results. Another limitation is that only six Doppler measurements were available in the early-onset group.

The lack of a replication cohort is a limitation. Therefore we used 10-fold cross validation to obtain more realistic performance estimates for the models. Initially our models reached quite promising AUC values for predicting pre-eclampsia but after validation the values decreased. A recent study using a combination of maternal risk factors, PAPP-A, PlGF, MAP and Uta-PI for predicting pre-eclampsia in the first trimester in a screening population also reported lower detection rates after five-fold cross validation [30] than in some earlier studies where there were not any kind of validation of the prediction rates [31, 32]. The reason for the modest prediction rates of our study compared to the studies conducted in low-risk or screening populations may be, that the role of impaired placentation could be less obvious in high-risk women, while maternal predisposing factors for vascular injury may become more important. It should be noted that high-risk conditions per se multiply the risk for pre-eclampsia.

The negative result of our attempt to increase the predictive power of a multivariate model with a new biomarker as well as the similar results from studies of others raises the question: Why are we not finding a good predictive model? Heterogeneous nature of pre-eclampsia poses a challenge. Myatt and co-workers [33] have suggested strategy to pre-eclampsia research including standardised data collection to hasten our understanding of the cause of the disease and to improve the early recognition.

## Conclusions

This study was a preliminary study investigating the potential of hCG-h or %hCG-h to improve the prediction of pre-eclampsia in a high-risk cohort in the first trimester using a multivariate regression model. There was a significant reduction of %hCG-h levels concentrations in women with late-onset and non-severe pre-eclampsia, but in combination with other biomarkers, maternal characteristics, MAP and Uta-PI, the sensitivity and the positive predictive values of the multivariate regression models did not meet the requirements for a clinically useful screening test among high-risk women.

## Additional files

Additional file 1: **Table S1.** Inclusion and exclusion criteria of the risk group. The inclusion and exlusion criteria for the risk group in PREDO project. (DOCX 67 kb)

Additional file 2: **Table S2.** The number of each inclusion criterion in women with and without pre-eclampsia. (DOCX 77 kb)

Additional file 3: **Table S3.** Concentrations of biomarkers, mean arterial pressure and uterine artery pulsatility index. The median concentrations of each biomarker, mean arterial pressure and uterine artery pulsatility index in women with and without pre-eclampsia and by pre-eclampsia subtype. (DOCX 78 kb)

## Abbreviations

%hCG-h: serum proportion of hCG-h to total hCG
AUC: area under the curve
BMI: body mass index
GA: gestational age in days
hCG: human chorionic gonadotropin
hCG-h: hyperglycosylated human chorionic gonadotropin
MAP: mean arterial pressure
MoM: multiple of the median
OR: odds ratio
PAPP-A: pregnancy-associated plasma protein A
PlGF: placental growth factor
PREDO: Prediction and Prevention of Pre-eclampsia and Intrauterine Growth Restriction cohort
ROC: receiver operating characteristic
SGA: small for gestational age
Uta-PI: mean pulsatility index of the uterine artery
