# Supplementary material for: Prediction of pre-eclampsia and its subtypes in high-risk cohort: hyperglycosylated human chorionic gonadotropin in multivariate models
Source: BMC Pregnancy Childbirth. 2018 Jul 3;18:279. doi: 10.1186/s12884-018-1908-9 (PMC6029382; doi:10.1186/s12884-018-1908-9)
Supplement: Supplementary file 1 — Table S1. Inclusion and exclusion criteria of the risk group. The inclusion and exlusion criteria for the risk group in PREDO project. (DOCX 67 kb) [file 12884_2018_1908_MOESM1_ESM.docx]

Table S1. Inclusion and exclusion criteria of the risk group

**Inclusion**

Obesity (body mass index over 30 kg/m2)

Chronic hypertension (≥140/90 mmHg or medication for hypertension before 20 weeks of gestation)

Sjögren’s syndrome

A history of Gestational diabetes

A history of pre-eclampsia (blood pressure ≥140 mmHg systolic or ≥90 mmHg diastolic
and proteinuria ≥0.3 g/day or dipstick equivalent in two consecutive measurements)

A history of small for gestational age (birthweight < 2SD)

A history of foetus mortus (foetal death after 22 weeks of gestation or >500 g weight in a previous pregnancy)

Systemic lupus erythematosus

Type I diabetes mellitus

**Exclusion**

Tobacco smoking (during this pregnancy)

Multiple pregnancy

A history of asthma

A history of peptic ulcer

Placental ablation

Inflammatory bowel diseases (Crohn’s disease, colitis ulcerosa)

Rheumatoid arthritis

Haemophilia or thrombophilia (previous venous or pulmonary thrombosis or coagulation abnormality)
