# Supplementary material for: Prediction of pre-eclampsia and its subtypes in high-risk cohort: hyperglycosylated human chorionic gonadotropin in multivariate models
Source: BMC Pregnancy Childbirth. 2018 Jul 3;18:279. doi: 10.1186/s12884-018-1908-9 (PMC6029382; doi:10.1186/s12884-018-1908-9)
Supplement: Supplementary file 2 — Table S2. The number of each inclusion criterion in women with and without pre-eclampsia. (DOCX 77 kb) [file 12884_2018_1908_MOESM2_ESM.docx]

Table S2. The number of each inclusion criterion in women with and without pre-eclampsia

| **Inclusion Criterion** | **Pre-eclampsia** | **No pre-eclampsia** | **p-value** | **OR** | **95 % Confidence Interval for OR**  **Lower Upper** | |
| --- | --- | --- | --- | --- | --- | --- |
| **Age under 20 years** | 1(2.9%) | 3 (1.3%) | 0.36 | 0.32 | 0.03 | 3.63 |
| **Age over 40 years** | 2(5.9%) | 27(12.1%) | 0.53 | 1.67 | 0.34 | 8.18 |
| **Obesity (body mass index over 30 kg/m2)** | 13(38.2%) | 85 (38.1%) | 0.70 | 0.84 | 0.35 | 2.04 |
| **Chronic hypertension (>140/90 mmHg or medication for hypertension before 20 weeks of gestation)** | 9(26.5%) | 41(18.4%) | 0.18 | 0.54 | 0.22 | 1.33 |
| **Type I diabetes mellitus** | 0 | 6 (2.7%) | NA* |  |  |  |
| **Sjögren’s syndrome** | 0 | 4 (1.8%) | NA* |  |  |  |
| **Systemic lupus erythematosus** | 0 | 1 (0.9%) | NA* |  |  |  |
| **A history of one of the following conditions:** |  |  |  |  |  |  |
| Gestational diabetes | 4 (11.8%) | 35 (15.7%) | 0.65 | 1.30 | 0.42 | 4.07 |
| Pre-eclampsia** | 16 (47.1%) | 64 (28.7%) | 0.04 | 0.39 | 0.16 | 0.97 |
| Small for gestational age (birthweight < 2SD) | 3 (8.8%) | 35 (17.5%) | 0.29 | 2.01 | 0.56 | 7.19 |
| Foetus mortus (foetal death after 22 weeks of gestation or >500 g weight in a previous pregnancy) | 1(2.9%) | 8(3.6%) | 0.98 | 0.98 | 0.11 | 8.62 |

* NA = not applicable, OR = Odds ratio

** Systolic blood pressure ≥140 mmHg, and/or diastolic blood pressure ≥ 90 mmHg occurring after 20 weeks of gestation in a woman with previously normal blood pressure, in combination with a urinary 24-hour protein excretion of > 0.3 g or dipstick equivalent in two consecutive measurements
