# Supplementary material for: Prediction of pre-eclampsia and its subtypes in high-risk cohort: hyperglycosylated human chorionic gonadotropin in multivariate models
Source: BMC Pregnancy Childbirth. 2018 Jul 3;18:279. doi: 10.1186/s12884-018-1908-9 (PMC6029382; doi:10.1186/s12884-018-1908-9)
Supplement: Supplementary file 3 — Table S3. Concentrations of biomarkers, mean arterial pressure and uterine artery pulsatility index. The median concentrations of each biomarker, mean arterial pressure and uterine artery pulsatility index in women with and without pre-eclampsia and by pre-eclampsia subtype. (DOCX 78 kb) [file 12884_2018_1908_MOESM3_ESM.docx]

| **Median / Mean** | **Valid cases PE/NO PE** | **PE** | **Women without PE** | **Early-onset PE n 9** | **Late-onset PE n 25** | **Severe PE n 17** | **Non-severe PE n 17** |
| --- | --- | --- | --- | --- | --- | --- | --- |
| **Median hCG, nmol/L (IQR)** | 34/223 | 61.1 (44.4) | 60.8 (38.6) | 85.6 (64.8) | 57.8 (39.3) | 68.8 (66.7) | 57.2 (35.4) |
| **Median hCG-h, nmol/L (IQR)** | 34/223 | 8.6 (10.1) | 9.4 (9.9) | 16.0 (16.5) | 7.8 (7.5) | 13.6 (18.1) | 7.8 (6.0) |
| **Median %hCG-h, % (IQR)** | 34/223 | 13.7 (10.3) | 15.9 (10.6) | 20.5 (12.0) | 12.4 (5.6) | 14.3 (14.4) | 12.1 (5.9) |
| **Median hCG-beta, ng/mL (IQR)** | 34/223 | 35.5 (27.5) | 29.8 (28.2) | 42.4 (19.9) | 31.8 (23.7) | 38.6 (40.0) | 34.5 (23.7) |
| **Median PAPP-A, mU/L (IQR)** | 34/223 | 1835 (1995) | 2140 (2290) | 1550 (820) | 2070 (2330) | 1720 (2420) | 1870 (2090) |
| **Median PlGF, ng/ml (IQR)** | 34/222 | 24.6 (12.5) | 27.0 (13.4) | 14.6 (8.5) | 27.3 (12.2) | 23.1 (17.9) | 24.8 (12.9) |
| **Median GW at sample taking (IQR)** | 34/223 | 13.14 (1.32) | 13.0 (0.86) | 12.86 (0.79) | 13.43 (1.43) | 13.0 (1.21) | 13.43 (1.36) |
| **Mean Uta PI (SD)** | 29/187 | 1.75 (0.47) | 1.54 (0.50) | 1.90 (0.28)* | 1.76 (0.51)** | 2.02 (0.80)# | 1.55 (0.66)## |
| **Median GW at US measurement (IQR)** | 29/187 | 12.71 (1.29) | 12.71 (0.86) | 12.57 (0.79)* | 13.00 (1.57)** | 12.57 (1.04)# | 13.00 (1.43)## |
| **Mean MAP, mmHg (SD)** | 34/211 | 102.1 (14.4) | 94.7 (10.9) | 104.3 (15.0) | 101.3 (14.5) | 105.8 (97.6-114.0) | 98.3 (92.1-104.5) |
| **Median GW at BP measurement (IQR)** | 34/211 | 12.74 (1.14) | 12.74 (1.43) | 12.57 (0.83) | 13.0 (1.43) | 12.57 (1.07) | 13.29 (1.57) |

Table S3. Concentrations of biomarkers, mean arterial pressure and uterine artery pulsatility index

PE = pre-eclampsia, GW=gestational weeks, BP=blood pressure, Uta = uterine artery, US=ultra sound, MAP= mean arterial pressure, PI=pulsatility index, IQR= interquartile range, SD= standard deviation.

* n=6, ** n=23, # n=14, ## n=15
